# Synthesis and Characterization of Biodegradable Polymers Based on Glucose Derivatives

**DOI:** 10.3390/ma16010253

**Published:** 2022-12-27

**Authors:** Barbara Gawdzik, Izabela Bukowska-Śluz, Anna E. Koziol, Liliana Mazur

**Affiliations:** Institute of Chemical Sciences, Faculty of Chemistry, Maria Curie-Sklodowska University, Gliniana 33, 20-614 Lublin, Poland

**Keywords:** glucose, acryl- and methacryl derivatives, copolymerization, biodegradable polymer, polymorphism of monomer

## Abstract

Syntheses of two new monomers, namely the glucose derivatives 2,3,4,6-tetra-*O*-acetyl-1 methacryloyl-glucopyranose (MGlc) and 2,3,4,6 tetra-*O*-acetyl-1-acryloylglucopyranose (AGlc), are presented. Their chemical structures were determined by the FTIR, ^1^H and ^13^C NMR spectroscopies, the single-crystal X-ray analysis, supported by the powder X-ray diffraction, and the DSC analyses. Molecules of both monomers exist in the β-anomeric form in the solid state. The variable temperature X-ray diffraction studies, supported by the DSC analyses, revealed AGlc’s propensity for polymorphism and temperature-induced phase transitions. MGlc and AGlc crystallised from methanol were polymerized or copolymerized with methyl methacrylate and N-vinylpyrrolidone. The biodegradabilities of polymers as well as thermal and optical properties were studied. The results show that some properties of the obtained homopolymers and copolymers resemble those of PMMA. The main difference is that the AGlc and MGlc homopolymers are biodegradable while PMMA is not. The ternary copolymers, i.e., MGlc/AGlc-MMA-NVP lose more than 10% of their weight after six months.

## 1. Introduction

Carbohydrates are the most abundant class of organic compounds found in living organisms. They are synthesised by green plants during the complex process of photosynthesis, in which sunlight provides the energy to transform carbon dioxide and water into glucose. Then, many glucose molecules are combined by covalent bonds, creating a reserve material in the form of plant cellulose or starch [1,2].

At present, sugars and their processed products are used in different areas of industry. Lately, they have been increasingly used for the production of plastic packaging [3]. Most plastic packaging is manufactured from synthetic polymers which are very durable and hardly decompose in the environment. This contributes to the increasing amount of plastic waste, causing serious environmental and economic problems. Therefore, the ideal solution to this problem is to produce plastics with biodegradable components [4,5,6,7,8].

Two approaches are usually used, involving the creation of blends with biodegradable fillers, for example, with starch, or research on the synthesis of polymers from new monomers obtained as a result of chemical modification of natural compounds [9,10].

Sugars are an attractive substrate for the synthesis of new polymers due to their abundance and availability. Incorporating them into complex chemical structures increases the likelihood of their disintegration.

However, polysaccharides are used for this purpose much more often than simple sugars. Among simple sugar-derived monomers, those containing bicyclic diols based on 1,4:3,6-dianhydrohexitols or diols derived from D-glucitol are studied extensively [11,12,13,14,15]. The D-glucose derivatives were also applied for the synthesis of potentially biodegradable ionic liquids, but within 20 days of the biodegradability test, no degradation was observed [16]. 

Generally, sugars are a subject of interest as materials with appropriate properties, such as good biocompatibility, high mechanical strength, safe degradation products, and natural bioactivity for the development of new materials [17,18].

This paper presents the synthesis and characterisation of meth/acrylic monomers— MGlc and AGlc—derived from the simple sugar, glucose. These compounds were mentioned in the scientific literature, but their synthesis and properties have never been described in detail [11]. The presence of the -OH group in the first position in the glucose molecule allowed the introduction of a double bond as a result of a reaction with unsaturated acids. Acrylic and methacrylic acids were used for the reaction. In this way, polymerizable glucose derivatives were obtained. 

Simultaneously, it is important to determine the stability of the molecules themselves, and the solid phase of monomers, used in chemical processes. Such factors as different solvents and temperatures used for crystallisation can result in different polymorphic phases, some of which can be spontaneously homopolymerized [19,20,21].

Using photopolymerization in the presence of Irgacure 651, transparent MGlc, and AGlc homopolymers and their copolymers with MMA and NVP monomers, which are precursors for creating polymers with significantly different biodegradability, were obtained. Transparent polymer films thus obtained could be potential packaging materials.

## 2. Experimental Section

### 2.1. Reagents

Glucose, phosphorus (red, amorphous), methacrylic acid, acrylic acid, and *N*,*N*-dimethylformamide were purchased from Fluka AG (Buchs, Switzerland). Reagent-grade potassium hydroxide, acetic anhydride, zinc chloride anhydrous, bromine, sodium bicarbonate, dichloromethane, methanol, cyclohexane, butyl acrylate, and styrene were purchased from POCh (Gliwice, Poland). Methyl methacrylate (MMA) and *N*-vinylpyrrolidone (NVP) were obtained from Sigma-Aldrich. Irgacure 651 (2,2-dimethoxy-1,2-diphenylethen-1-one) was purchased from Ciba (Basel, Switzerland).

### 2.2. Synthesis of Monomers (MGlc and AGlc)

#### 2.2.1. Preparation of Potassium Meth/Acrylate

A total of 500 mL of dichloromethane, 20 mL of water and 33 g of potassium hydroxide were placed in a flask and cooled to 0 °C. At this temperature, 0.5 moles of methacrylic acid (43 g) or 36 g of acrylic acid was added while stirring vigorously. A large exothermic effect caused the dropping rate to be controlled in order to maintain a temperature not higher than 15 °C. At this temperature, stirring was continued for 1 h. The obtained precipitate of meth/acrylate potassium salt was filtered off and dried in air (yield ~83%).

#### 2.2.2. Preparation of 1-bromo-2,3,4,6-tetra-O-acetyl-D-glucopyranose

In the synthesis of both monomers, there was used the same starting compound i.e., bromide 2,3,4,6-tetra-*O*-acetyl-D-glucopyranose. To obtain it the procedure described by Vogel was applied [22]. According to this procedure, 2.12 mole (200 mL) of acetic anhydride, and 8 g of zinc chloride anhydrous were placed in a three-necked flask and stirred till zinc chloride anhydrous dissolved. Next, 45 g (0.25 mole) of glucose was added in portions while stirring. The temperature did not exceed 30 °C. After adding the whole amount of glucose, the mixture was heated for 1 h over the water bath. When the reaction was over, the mixture was cooled to 20 °C, and 0.5 moles (15.5 g) of phosphorus (red, amorphous) was added while stirring. Next, 29 mL (0.56 mole) of bromine was dropped while stirring so that the temperature did not exceed 20 °C. After adding bromine, 18 mL of water was added, and the mixture was kept at room temperature for 24 h. Then it was diluted with 150 mL of dichloromethane and filtered off. The organic layer was transferred to the distributor and washed with two portions of iced water. Then the organic layer (dichloromethane solution) was added while stirring into a saturated sodium bicarbonate solution containing a bit of crushed ice. When carbon dioxide ceased to exude, the organic layer was dried over the silica gel and the solvent was removed on a rotary evaporator at 60 °C. 

The obtained 90 g of 1-bromo-2,3,4,6-tetra-*O*-acetyl-D-glucopyranose precipitate was dried in the air. After crystallising from cyclohexane, its mp was 88–89 °C. The chemical structure was confirmed by the elemental, ^1^HNMR, and FTIR analyses.

#### 2.2.3. Preparation of 2,3,4,6-tetra-O-acetyl-1-methacryloylglucopyranose (MGlc)

To obtain MGlc 8.5 g of 1-bromo-2,3,4,6-tetra-*O*-acetyl-D-glucopyranose, 7.5 g of potassium methacrylate and 32 mL of *N*,*N*-dimethylformamide (solvent) were placed in a flask, and heated to 65 °C while stirring. This temperature was maintained for 30 minutes. When the reaction was over, the resulting mixture was poured into the water containing crushed ice while stirring constantly. Next, the obtained MGlc precipitate was filtered off and dried in air. The crystallisation from methanol yielded 6.43 g of this compound (~79%) with mp 100.0 °C, and after crystallising from cyclohexane, mp was 92.9 °C. 

Elem. anal.: calcd. for C_18_H_24_O_11_: C, 51.92; H, 5.80; O, 42.28. Found: C, 51.88; H, 5.80; O, 42.32. ^1^HNMR (300 MHz, CDCl_3_, δ, ppm): 1.95 (s, 3H), 2.05 (t, J1 = 2.52 Hz, J2 = 2.52 Hz, 9H), 2.08 (s, 3H), 3.90 (dc, J1 = 2.25 Hz, J2 = 2.23 Hz, J3 = 2.24 Hz, J4 = 2.24 Hz, 1H), 4.14 (dd, J1 = 2.21 Hz, J2 = 2.21 Hz, 1H), 4.32 (dd, J1 = 4.46 Hz, J2 = 4.47 Hz, 1H), 5.25 (m, 3H), 5.70 (m, 1H), 5.76 (d, J1 = 7.25 Hz, 1H), 6.20 (m, 1H). ^13^CNMR (75 MHz, CDCl_3_, δ, ppm): 18.10 (-CH_3_), 20.66 (-CO-CH_3_), 61.57 (-CH_2_-), 128.35 (CH_2_=), 134.90 (-C=), 165.18 (-CO-O-).

#### 2.2.4. Preparation of 2,3,4,6-tetra-O-acetyl-1-acryloylglucopyranose (AGlc)

To obtain AGlc, a procedure similar to that described earlier for MGlc was applied. In this case, 8.5 g of 1-bromo-2,3,4,6-tetra-*O*-acetyl-D-glucopyranose, 6.4 g of potassium acrylate, and 32 mL of *N*,*N*-dimethylformamide (solvent) were mixed and heated up to 65 °C while stirring. When the obtained AGlc crystallised from methanol, 4.60 g of this compound was obtained (yield ~72%), and its mp was 109.2 °C, whereas that which was determined after the crystallisation from cyclohexane was 106.3 °C. 

Elem. anal.: calcd. for C_17_H_22_O_11_: C, 50.75; H, 5.51; O, 43.74. Found: C, 50.58; H, 5.49; O, 43.93. ^1^HNMR (300 MHz, CDCl_3_, δ, ppm): 2.05 (t, J1 = 1.87 Hz, J2 = 1.90 Hz, 9H), 2.08 (s, 3H), 3.85 (dc, J1 = 2.24 Hz, J = 22.25 Hz, J3 = 2.24 Hz, J4 = 2.25 Hz, 1H), 4.12 (dd, J1 = 2.19 Hz, J2 = 2.21 Hz, 1H), 4.30 (dd, J1 = 4.50 Hz, J2 = 4.50 Hz, 1H), 5.25 (m, 3H), 5.79 (d, J1 = 7.96 Hz, 1H), 5.95 (dd, J1 = 1.32 Hz, J2 = 1.31 Hz, 1H), 6.12 (dd, J1 = 10.48 Hz, J2 = 10.50 Hz, 1H), 6.50 (dd, J1 = 1.31 Hz, J2 = 1.31 Hz, 1H). ^13^CNMR (75 MHz, CDCl_3_, δ, ppm): 20.64 (-CO-CH_3_), 61.54 (-CH_2_-), 127.14 (CH_2_=), 133.59 (-C=), 163.86 (-CO-O-).

### 2.3. Characterization

#### 2.3.1. Methods for Monomers

The attenuated total reflectance Fourier transform infrared (ATR-FTIR) spectra were obtained with a Bruker TENSOR 27 spectrometer equipped with a diamond crystal (Bruker, Germany). The spectra were gathered from 600 to 4000 cm^−1^ with 32 scans per spectrum at a resolution of 4 cm^−1^. The ^1^H NMR and ^13^C NMR spectra were recorded with a Bruker Avance 300 MHz instrument (Bruker, Germany) in deuterated chloroform. Chemical shifts were reported regarding chloroform (δ 7.26) for ^1^H NMR, and chloroform (δ 77.28) for ^13^C NMR. The elemental analysis of the product was performed using Perkin Elmer CHN 2400. Melting points (peak temperature) of the MGlc and AGlc monomers were determined by the differential scanning calorimetry (DSC) measurements using a Netzsch DSC 204 (Netzsch, Selb, Germany). The samples of about 10 mg weight were placed in an aluminium pan with a pierced lid, put into the DSC oven, and heated up to 150 °C with the heating rate of 5 °C min^−1^. The measurements were made under the dynamic argon atmosphere (20 mL min^−1^). The empty pan was used as a reference sample.

#### 2.3.2. X-Ray Crystallography

Single crystals of MGlc and AGlc, suitable for the X-ray diffraction studies, were grown by recrystallization from dry methanol, cyclohexane, or cyclohexane—acetone (3:1, *v*/*v*) mixture, using the standard solvent evaporation technique. The diffraction data for AGlc-**M**, AGlc-**M**-**LT** were collected on the SuperNova diffractometer (Oxford Diffraction) equipped with the microfocus X-ray source (Cu Kα, λ = 1.54184 Å) and the CCD detector. The single-crystal X-ray measurements on MGlc and AGlc-**O** were conducted using the Rigaku XtaLAB MM7HFMR diffractometer equipped with the “quarter-chi single” goniometer, the rotating anode generator (graphite monochromated Cu Kα radiation), and the Pilatus 200K detector. The CrysAlisPro 1.171.39.27b program was used for data collection, cell refinement, and data reduction [23]. The intensities were corrected for the Lorentz and polarisation effects. The multi-scan absorption correction was applied. The structures were solved using the direct methods implemented in the *SHELXS-97* and refined with the *SHELXL-18/3* program, both operating under *WinGX* [24,25]. Non-H atoms were refined with anisotropic displacement parameters, except for AGlc-**M**-**LT**, where a partial disorder was observed. The hydrogen atoms were positioned geometrically and refined using the riding model with *U_iso_*(H) = 1.2*U_eq_*(CH and CH_2_) or *U_iso_*(H) = 1.5*U_eq_*(CH_3_).

CCDC Nos 2218910–2218913 contain the supplementary crystallographic data for MGlc and AGlc. The copies of the data can be obtained free of charge on application to CCDC, 12 Union Road, Cambridge CB21EZ, UK (Fax: +44-1223-336-033; e-mail: deposit@ccdc.cam.ac.uk or www: http://www.ccdc.cam).

#### 2.3.3. X-Ray Powder Diffraction

The powder X-ray diffraction data were collected using the Panalytical Empyrean diffractometer with Ni-filtered Cu Kα radiation (Malvern Panalytical, Malvern, UK). The intensity data were captured with the PIXcel^3D^ detector over the 2θ range of 5−50°. We used two modes of measurement: the standard at room temperature measurement, and the second measurement in the reactor chamber at variable temperatures. In the first method of measurement, the step was Δθ = 0.013° while in the second mode Δθ = 0.026°. The overlays of diffraction patterns were generated using Highscore software [26].

### 2.4. Polymerization

Polymerisation of the obtained meth/acrylate glucose derivatives was conducted by radical polymerisation using the photoinitiator Irgacure 651. To avoid the instability of the reagents (due to polymorphism) during the polymerisation process, only the monomers MGlc and AGlc crystallised from methanol were used. Copolymers were obtained by dissolving 4 g of the compounds in 16 g of MMA, or in 16 g of the mixture of MMA and NVP 1:1 (*w*/*w*) (Table 1). Next, 1 % *w*/*w* of Irgacure 651 was added, and the solution was photopolymerised with the use of UV lamp [27,28]. Photopolymerisation was performed with the use of a commercially available Blacklight lamp 366 nm (Philips, TSM0022 TL-D18W) at room temperature. The width of all obtained foils was 4 mm. The irradiated samples were distanced 15 cm from the lamp. The process was carried out for 3 h. According to the same procedure, homopolymers of AGlc, MGlc, copolymer MMA-NVP, and PMMA as reference material were prepared. 

### 2.5. Optical Properties

The ultraviolet-visible (UV/Vis) spectra of MGlc and AGlc homopolymers and their copolymers with MMA were measured by the UV-2550 spectrophotometer (Shimadzu, Kioto, Japan) in the range of 200–900 nm, and at a scanning rate of 200 nm min^−1^. The analysed samples were in solid form (bulk polymers) of 3 mm thickness.

### 2.6. Thermal Stability Studies of Polymers

To determine thermal stabilities of polymeric samples, thermogravimetric measurements were made using the STA 449 Jupiter F1, Netzsch (Selb, Germany) under the following operational conditions: the heating rate 10 °C min^−1^, the dynamic atmosphere of helium (50 mL min^−1^) in the temperature range of 30–500 °C, the sample mass of about 5 mg, the sensor thermocouple type S TG-DSC. As a reference, an empty Al crucible was used. The initial decomposition temperatures (IDT) were determined from the course of the TG curves.

### 2.7. Biodegradation Test

Biodegradation of the obtained polymers was studied according to the accelerated soil burial test [29,30]. Three samples of each polymeric foil were cut to reach the sizes 60–65 mm × 28–30 mm. The samples were buried in the biologically active soil free of impurities and particles (>10 mm) at the depth of ca. 20 mm. The soil was maintained at pH 5–7, a relative humidity of 19–21%, and a temperature of 22 ± 2 °C. The tested specimens were extracted every 30 days for 180 days. They were cleaned, dried in a vacuum dryer at 50 °C for 5 h, and then they were left for 1 day in a desiccator to ensure water desorption before measurements. To determine the mass loss, the samples were weighed.

## 3. Results and Discussion

### 3.1. Synthesis of Monomers

The paper presents new monomeric derivatives of glucose meth/acrylate (MGlc and AGlc). It turned out that in the preparation of meth/acrylate glucose derivatives, direct esterification with acids, which we used many times, was ineffective or even impossible [31,32,33]. The most effective was the procedure based on 1-bromo-2,3,4,6-tetra- O-acetyl-D-glucopyranose and further reaction of the bromo-derivative of glucose obtained in this way with a salt of acrylic or methacrylic acid. The recipe described by Vogel was used for the synthesis of 1-bromo-2,3,4,6-tetra-O-acetyl-D-glucopyranose and the product was obtained in good yield (~90%). 

Then the compound was transferred into MGlc and AGlc using the new method proposed by us, based on the reaction with a potassium salt of methacrylic or acrylic acid in *N*,*N*-dimethylformamide. This procedure allowed to obtain meth/acrylate derivatives of the naturally occurring monosaccharide with a satisfactory yield (~80%).

**Scheme 1:**
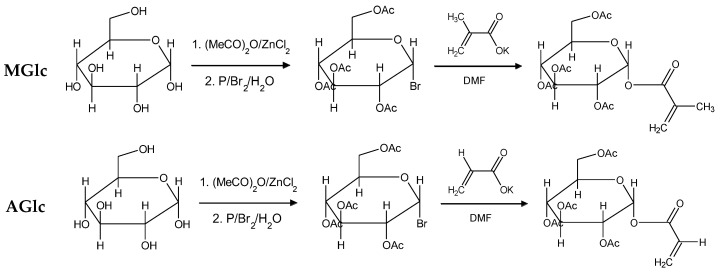
Synthesis of new derivatives of glucose: **MGlc**—2,3,4,6-tetra-*O*-acetyl-1-methacryloylglucopyranose and **AGlc**—2,3,4,6-tetra-*O*-acetyl-1-acryloylglucopyranose.

The syntheses of the obtained glucose derivatives are presented in Scheme 1. Both AGlc and MGlc are white, crystalline compounds. They are insoluble in water but well soluble in organic solvents such as methanol, cyclohexane, and acetone. The characterisation of the physicochemical properties of these compounds was made using elemental and thermal analyses, spectroscopic methods, and powder X-ray diffraction. Molecular and crystal structures were determined by a single-crystal X-ray crystallography.

### 3.2. Thermal and X-ray Diffraction Studies of Solid Monomers

**Figure 1:**
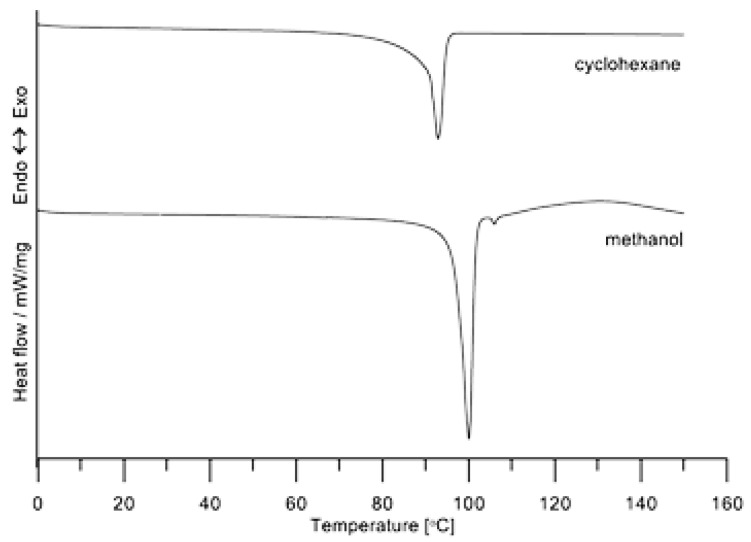
DSC thermograms of MGlc.

**Figure 2:**
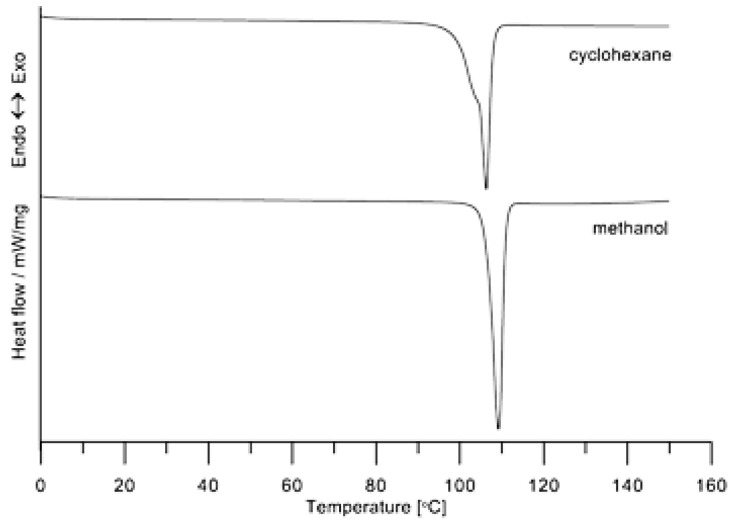
DSC thermograms of AGlc.

**Figure 3:**
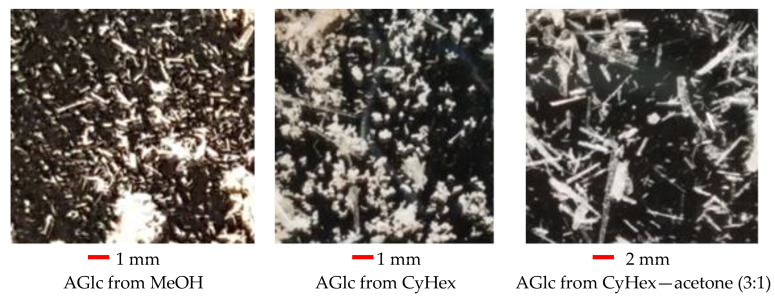
Crystal morphology observed for AGlc crystallised from the solvents: methanol, cyclohexane, and the mixture of cyclohexane–acetone (v:v; 3:1).

An interesting fact observed for these compounds is the existence of two melting points, depending on the solvent used for crystallisation viz. methanol or cyclohexane. The sample of MGlc obtained by the crystallisation from methanol melts at 100.0 °C, while recrystallisation from cyclohexane determines the product of the melting point at 92.9 °C (Figure 1). Similar behaviour was observed for solid AGlc; the melting point was at 109.2 °C for the sample crystallised from methanol, while after recrystallisation from cyclohexane, it was 106.3 °C (Figure 2). Interestingly, independent of the solvent used, recrystallisation of AGlc via slow solvent evaporation resulted in the growth of very thin needle-like crystals of a similar size and morphology (Figure 3).

**Figure 4:**
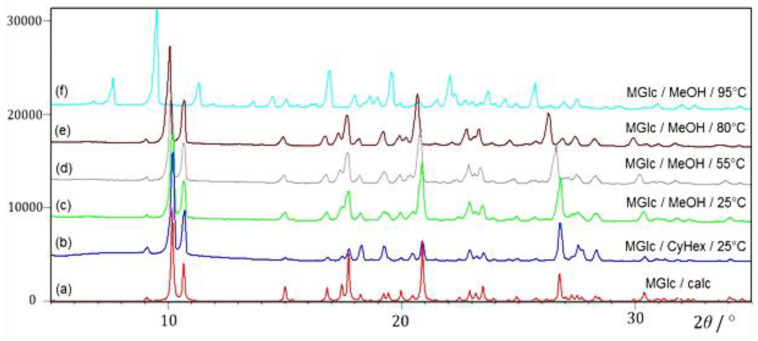
Powder X-ray diffraction patterns of the samples of MGlc crystallised from cyclohexane (**b**) and from methanol (**c**–**f**), were recorded at 25, 55, 80, and 95 °C. The reference pattern of MGlc (**a**) calculated from single-crystal data is given in red.

Therefore, taking into account the different melting points, comprehensive single-crystal and powder X-ray diffraction studies were undertaken. As follows from those data (Figure 4), the recrystallization of MGlc from the methanol and cyclohexane solutions gave solely the monoclinic chiral crystals (the space group *P*2_1_, Table 2). For MGlc, we observed the complex thermodynamic behaviour, depending on the experimental method used. The X-ray powder patterns, recorded at room temperature for the samples obtained from methanol and cyclohexane, are almost identical and comparable with those calculated from the single-crystal structural data (Figure 4a–c). However, the DSC results revealed significant differences in their melting point as well as in the course of the melting process (Figure 1).

**Figure 5:**
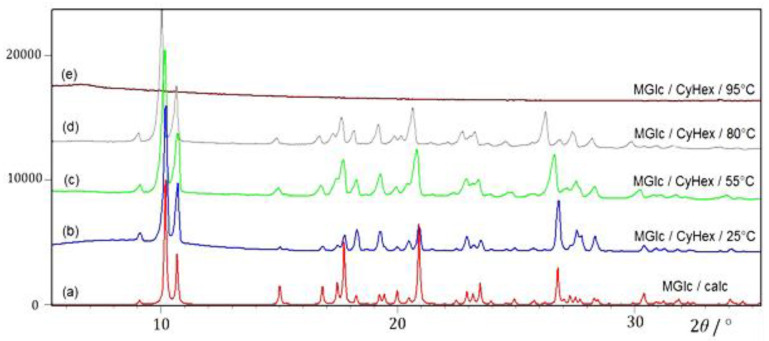
Powder X-ray diffraction patterns of MGlc crystallised from cyclohexane were recorded at 25, 55, 80, and 95° C (**b**–**e**). The reference pattern of MGlc (**a**) calculated from the single-crystal data is given in red.

In order to verify the thermal stability of the samples, the diffraction experiments in the closed chamber at variable temperatures were performed using a bulk material obtained by recrystallization of MGlc from methanol and cyclohexane (Figure 4 and Figure 5). 

A great similarity was found for MGlc between the appropriate powder patterns recorded at 55 and 80 °C (Figure 4d,e and Figure 5b–d). However, at higher temperatures, the thermal behaviour of the batches was completely different. After heating the samples to 95 °C the MGlc (from CyHex) was completely melted (Figure 5e) whereas the sample MGlc (from MeOH) underwent the phase transition as confirmed by the shift and appearance/disappearance of many diffraction peaks characteristic of MGlc (Figure 4f). Absorption of undetectable amounts of solvent between the blocks of MGlc crystallites is possible, which affects the thermal stability of the entire phase. Thus, comparing the above diffraction data with the results of the DSC analysis performed in the dynamic atmosphere of argon (Figure 1), it can be concluded that for MGlc (from MeOH) the melt-recrystallization phase transition occurs prior to the final melting.

**Figure 6:**
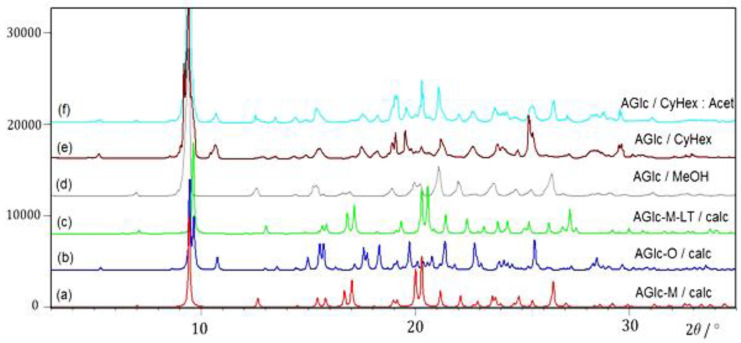
Powder X-ray diffraction patterns of AGlc recrystallised from methanol (**d**), cyclohexane (**e**), and the cyclohexane–acetone (3:1) mixture (**f**). The reference patterns of polymorphic forms AGlc-**M**, AGlc-**O**, and AGlc-**M**-**LT** (calculated from data from the X-ray structure analysis) are given in red, dark blue, and green, respectively (**a**–**c**).

In the case of AGlc, different products and effects were observed from those for MGlc. For AGlc, two polymorphs (**M,** monoclinic and **O**, orthorhombic) were found in the resulting crystalline batches. The structure of AGlc form depends on the polarity of the solvent used in the crystallisation process. Differences in intermolecular interactions between AGlc and solvents (methanol or cyclohexane) affect both nucleation and crystal growth. Thus, the form AGlc-**M** obtained from dry methanol crystallises in the monoclinic space group *P*2_1_ with one molecule in the asymmetric unit of the crystal (Table 2; Figure S1, Supporting Information), while the recrystallization from cyclohexane led to the orthorhombic form AGlc-**O**, the space group *P*2_1_2_1_2_1_, with two molecules in the asymmetric part of the unit cell (Figure S2). Notably, the experimental powder patterns of AGlc-**M** and AGlc-**O** were in very good agreement with those calculated from the single-crystal X-ray data, which confirmed the phase purity of the bulk samples (Figure 6).

The combined DSC (Figure 2) and single-crystal diffraction data revealed that the polymorph AGlc-**O** is stable in a wide range of temperatures, and it does not undergo any changes from 150 °C up to the melting point (about 106 °C). In the case of the polymorph, no AGlc-**M** peaks on the DSC curves (recorded from 0 °C to 150 °C) were observed (Figure 2) which excluded any thermal effects prior to melting. However, after cooling the AGlc-**M** crystals down to –153 °C in the single-crystal X-ray diffraction experiments, a change in the unit cell parameters was observed. Thus, the new low-temperature polymorphic phase AGlc-**M**-**LT** was formed (Figure S3, Supplementary Materials).

Further studies confirmed the reversible crystal-to-crystal (AGlc-**M** ↔ AGlc-**M**-**LT**) phase transition in the temperature range of 210–240 K (from −63 °C to −33 °C). The change of the crystal lattice parameters during conversion to the form AGlc-**M**-**LT** (the space group *P*2_1_, Z’ = 3) resulted from the conformational adjustment, accompanied by some modifications of the molecular arrangement in the crystal (Figures S1 and S3, Supplementary Materials). Such a phase transformation is possible due to the presence of only weak intermolecular interactions of the C–H^...^O type in the crystal net (Figure S4). The detailed analysis of conformers and crystal structures is given in the Supplementary Information file.

**Figure 7:**
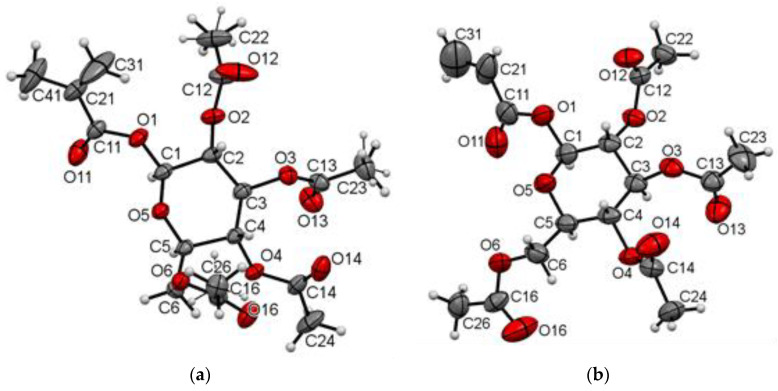
Perspective view of molecules: (**a**) MGlc and (**b**) AGlc-**M**. The thermal ellipsoids are shown at a 30% probability level.

The crystallographic data for all detected crystal forms of MGlc and AGlc are summarised in Table 2. All crystals adopt the non-centrosymmetric space groups. The molecular plots for the studied compounds with the atom-labelling schemes are presented in Figure 7. The selected geometric parameters are given in Table S1.

As follows from the structural data, the molecules of both compounds exist as the β-anomers in a solid state (Figure 7). In all structures, the pyranose ring has a slightly distorted (^4^C_1_) chair conformation. The equatorial position of the meth/acryl group is confirmed by the O1–C1–O5–C5 torsion angle, which is approximately 180° (±8°). The orientation of the acetoxy groups at the ring positions C2, C3 and C4 as well as the substituent at C5 are also equatorial (Table S1).

**Figure 8:**
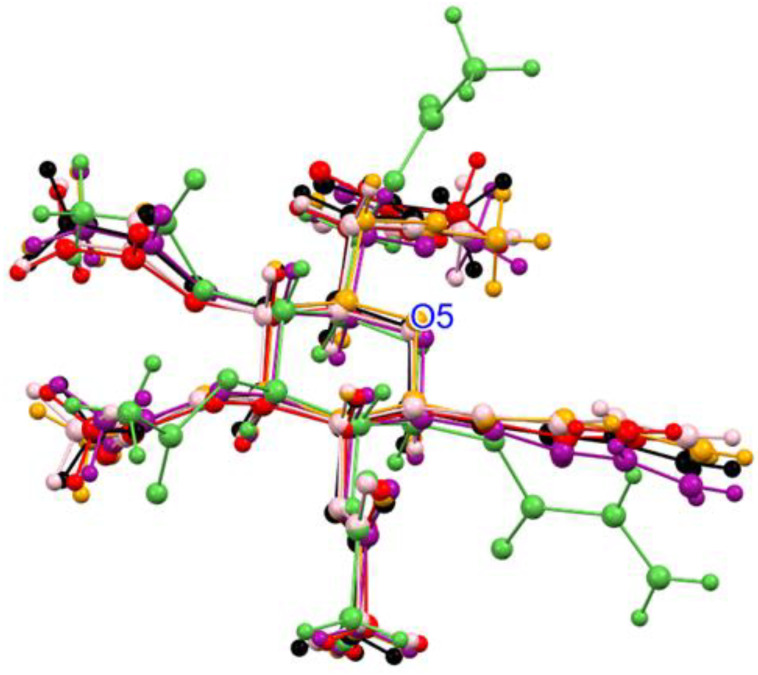
The overlay of the AGlc conformers present in the asymmetric part of the unit cell in the crystals: AGlc-**M** (black line), AGlc-**O** (molecule A—green line and B—violet line), and AGlc-**M**-**LT** (molecule A—pink line, B—red line, and C—orange line).

Due to polymorphism, the six solid-state conformers are examined for the AGlc molecule. The molecular overlay of AGlc-**M**, AGlc-**M**-**LT,** and AGlc-**O** (Figure 8) indicates that the essential difference between the conformers in the polymorphs is in the relative orientation of the acryl and acetyl groups with respect to the central pyranose. That is largely due to the rotation around the C–O bonds (ring carbon–ester oxygen). Significant conformational differences between the molecules AGlc-**M**, AGlc-**M**-**LT,** and AGlc-**O**, described by the values of the torsion angles (Table S1, Supplementary Materials), suggest the detection of conformational polymorphism [34]. The most significant changes are observed for the torsion angles O5–C5–C6–O6, O5–C1–O1–C11, C1–C2–O2–C12, C2–C3–O3–C13, C5–C6–O6–C16, for which respective differences are up to Δτ ≈ 115°.

### 3.3. FT-IR Spectroscopy

**Figure 9:**
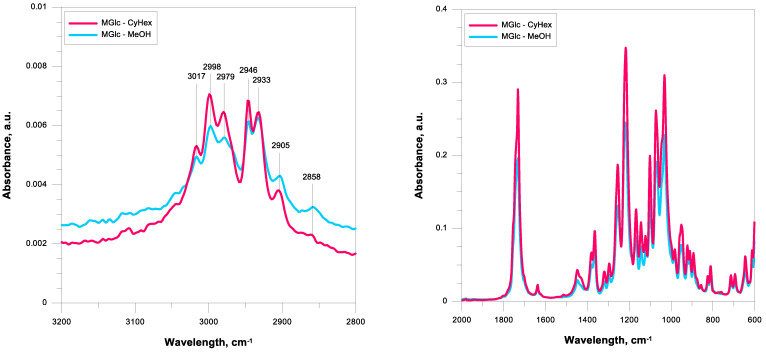
FTIR spectra of MGlc monomer crystallised from methanol (blue line), and cyclohexane (red line).

**Figure 10:**
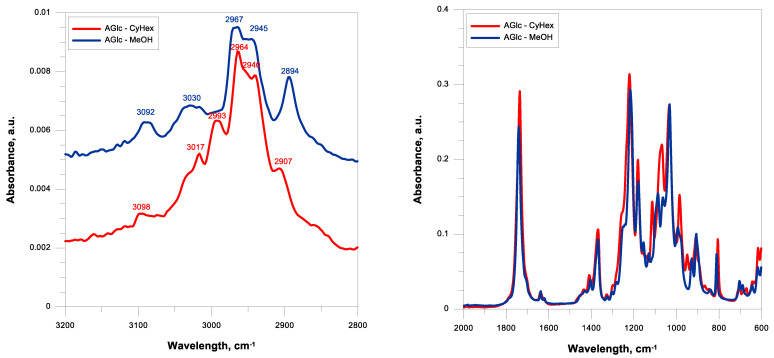
FTIR spectra of AGlc monomer crystallised from methanol (blue line), and cyclohexane (red line).

Polymorphism for AGlc was also confirmed by the results of ATR-FTIR analysis. The spectra of AGlc crystallised from methanol and cyclohexane show significant differences, while the spectra for the MGlc samples obtained from the above-mentioned solvents differ only in intensities. According to Ibrahim et al., characteristic bands for carbohydrates are present in the region 600–1500 cm^−1^ representing the C–O and C–C vibrations [35]. In both spectra (Figure 9 and Figure 10), a typical peak of glucose at 1033 cm^−1^ is observed. For AGlc crystallised from methanol, the additional peaks are at 920, 1130, 1282, 1621 cm^−1^, and two peaks at 1064 and 1087 cm^−1^, instead of 1069 cm^−1^, present in the spectrum of AGlc crystallised from cyclohexane. On the spectrum of the sample AGlc (CyHex), two additional peaks of low intensities at 870, 949 cm^−1^ are visible. 

In the wavenumber range from 2900 to 3200 cm^−1^, differences between the spectra for AGlc and MGlc are also observed. For MGlc, the spectra of the samples crystallised from methanol and cyclohexane show no differences (Figure 9). One can see the bands with high intensity at 2933, 2946, 2979, and 2998 cm^−1^ and three low-intensity bands at 2858, 2905, and 3017 cm^−1^.

In contrast, the spectra of AGlc are significantly different from MGlc. Moreover, there are noticeably different spectra for the AGlc samples crystallised from methanol and cyclohexane (Figure 10). For the crystals from methanol, well-visible bands are at 2894 and for the wide ones, the well-visible bands are at 2945, 2967, and 3092 cm^−1^ (in the range 3010–3050 cm^−1^). For the sample AGlc, (from CyHex) there is no peak at 2894 cm^−1^ but the bands at 2907, 2993 and 3017 cm^−1^ observed for the MGlc were visible. There are also bands at 2940, 2964, and at 3098 cm^−1^ assigned to the C–H bending.

### 3.4. Polymerization

The terminal meth/acrylate double bonds in the obtained compounds were used for polymerisation. The solubility of MGlc and AGlc in the solvents, often used as monomers in copolymerisation, was determined. Both are very well soluble in methyl methacrylate (MMA), *N*-vinylpyrrolidone (NVP), butyl acrylate (AB), and styrene, forming transparent polymers. Using the Irgacure-initiated photopolymerization, the homopolymers of MGlc, AGlc, and their copolymers with MMA and NVP were obtained (Table 1).

### 3.5. Optical Properties

To evaluate the transparency of MGlc and AGlc co/polymers, their transmittances were measured. The obtained values were compared with that for PMMA measured under the same conditions (Table 3). The results show that at 800 nm the values of transmittance of MGlc and AGlc polymers exceed 80%, and are comparable to that of PMMA; the AGlc homopolymer shows even greater transmittance. At the wavelength of 400 nm, near UV light, the homopolymers of MGlc and AGlc have a much lower transmission than PMMA. The transmittance for MMA exceeds 80% while that for AGlc is 59.4%, and it does not even reach 50% for MGlc. Obviously, the copolymers with MMA show better transparency. The poorer transparency of MGlc and AGlc polymers in this light range is due to the presence of glucose moieties showing the absorption of UV radiation [36].

### 3.6. Thermal Properties of Polymers

The thermal properties of polymers and copolymers with MMA and NVP obtained in the photopolymerization process are summarised in Table 4. The homopolymers of MGlc and AGlc are the most thermally resistant. Their initial decomposition temperatures (IDT) are 142.6 °C and 179.3 °C, and both are higher than that for PMMA which is equal 127.0 °C. In the case of copolymers with MMA, one can see that MGlc-MMA has a higher IDT (146.3 °C) than PMMA, whereas for AGlc-MMA (133.2 °C) it is close to that of PMMA. The addition of NVP caused the IDT of MGlc-MMA-NVP and AGlc-MMA-NVP to become significantly lower (but higher than that for the MMA-NVP copolymer). The thermal degradation process for the copolymers with NVP has a different course. In these cases, two maxima on the course of their degradation were observed.

### 3.7. Biodegradability of Polymers

**Figure 11:**
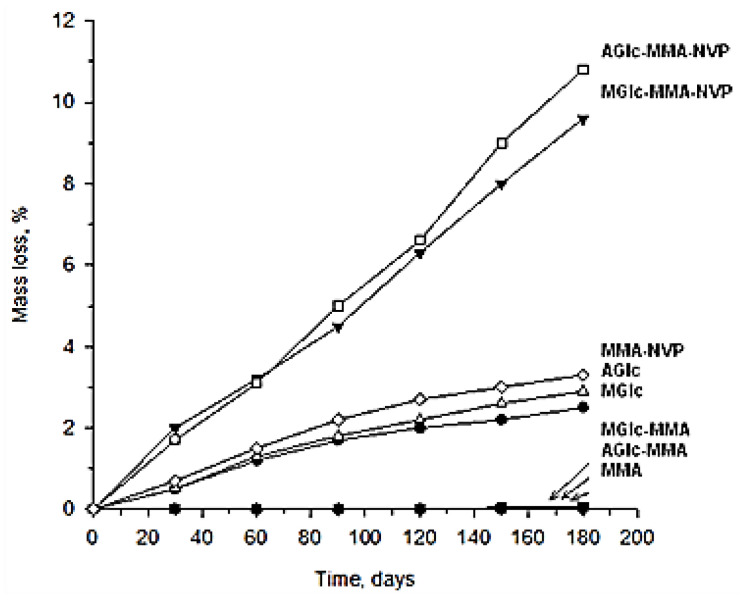
Results of biodegradation studies: mass loss (%) versus time (days).

Soil tests indicate that the homopolymers of both MGlc and AGlc are biodegradable. Their weight loss after 180 days of the sample presence in soil is in the range of 2.5–2.9% (Figure 11). The introduction of MGlc and AGlc into the structure of the MMA copolymer initiated its degradation. The weight loss for MGlc-MMA and AGlc-MMA was 0.02 and 0.06%, respectively, while PMMA alone is not biodegradable at all. The addition of the hydrophilic monomer NVP caused a significant acceleration of degradation. After 180 days, the weight loss of the copolymers MGlc-MMA-NVP and AGlc-MMA-NVP was about 10%. This means that the addition of MGlc or AGlc monomers can cause the biodegradation of absolutely non-biodegradable polymers such as PMMA.

## 4. Conclusions

The results presented here show that glucose can be easily transferred into meth/acrylic monomers. The results of X-ray crystal structure and FT-IR analyses confirm that both AGlc and MGlc crystallised from methanol as the β-anomers. 

Photopolymerization of these compounds, alone or together with MMA and NVP, leads to the formation of transparent copolymers. The thermal resistance of the obtained AGlc and MGlc homopolymers/copolymers is higher than that of PMMA. The biodegradation studies of the polymers indicate that under conditions imitating nature, they undergo slow decomposition. The addition of NVP caused the initial decomposition temperature of MGlc-MMA-NVP and AGlc-MMA-NVP to become significantly lower. Simultaneously, the biodegradation of these copolymers accelerates significantly. In some applications, the newly obtained homopolymers can compete with PMMA. In comparison with PMMA, they show a higher thermal resistance and comparable transparency, and they are biodegradable.

## Figures and Tables

**Table 1:** Chemical composition (%) of the glucose-derived polymers.

Polymer	MGlc	AGlc	MMA	MMA-NVP (1:1)
MGlc	100			
MGlc-MMA	20		80	
MGlc-MMA-NVP	20			80
AGlc		100		
AGlc-MMA		20	80	
AGlc-MMA-NVP		20		80
PMMA			100	
MMA-NVP				100

**Table 2:** Crystal data and selected structure refinement details for the monomers MGlc and AGlc (polymorphs **M**, **O**, **M**-**LT**).

Crystal Structure	MGlc	AGlc-M	AGlc-O	AGlc-M-LT
Solvent used	methanol	methanol	cyclohexane	
Experimental T/K	293	293	293	120
Chemical formula	C_18_H_24_O_11_	C_17_H_22_O_11_	C_17_H_22_O_11_	C_17_H_22_O_11_
Formula weight	416.37	402.34	402.34	402.34
Crystal system	monoclinic	monoclinic	orthorhombic	monoclinic
Space group	*P*2_1_	*P*2_1_	*P*2_1_2_1_2_1_	*P*2_1_
*a*/Å	9.729(1)	5.821(1)	5.976(1)	14.813(4)
*b*/Å	6.264(1)	13.970(3)	18.730(2)	13.566(6)
*c*/Å	17.351(2)	12.769(3)	36.605(4)	15.550(5)
*β*/°	93.24(1)	100.06(2)	90	110.08(4)
*V*/Å^3^	1055.7(2)	1022.4(4)	4097.2(9)	2935(2)
*Z/Z’*	2/1	2/1	8/2	6/3
*d*_calc/_g·cm^−3^	1.310	1.307	1.305	1.366
*µ*/mm^−1^	0.943	0.956	0.954	0.999
*R*_int_	0.0633	0.015	0.0661	0.0527
Reflections indep./with *I* > 2*σ*(*I*)	3871/3711	2897/2301	7277/4213	8257/4234
R_1_ [*I* > 2*σ*(*I*)]	0.0350	0.0977	0.0558	0.2165
*w*R_2_ [all data]	0.1007	0.3034	0.1767	0.5654
min/max [e·Å^−3^]	−0.11/0.14	−0.27/0.53	−0.18/0.31	−0.70/1.48
CCDC No.	2218910	2218911	2218912	2218913

**Table 3:** Optical properties of homopolymers of MGlc and AGlc, and copolymers with MMA. Transmittance at 400 and 800 nm, respectively.

Polymer	T_400_	T_800_
MGlc	48.9	81.0
AGlc	59.4	88.7
MGlc-MMA	71.4	85.1
AGlc-MMA	71.0	88.7
PMMA	83.0	86.0

**Table 4:** Thermal properties (°C) of the obtained polymers.

Polymer	IDT	T_end_	T_max1_	T_max2_
MGlc	142.6	536.9	280.2	
AGlc	179.3	573.5	277.9	
PMMA	127.0	403.0	292.5	
MGlc-MMA	146.3	502.4	296.7	
MGlc-MMA-NVP	74.5	652.4	303.7	412.8
AGlc-MMA	133.2	625.3	303.7	
AGlc-MMA-NVP	78.5	642.6	302.3	421.1
MMA-NVP	52.3	630.8	412.4	

## Data Availability

No data was used for the research described in the article.

## Supplementary Materials

The following supporting information can be downloaded at https://www.mdpi.com/article/10.3390/ma16010253/s1, Figure S1: Molecular packing in crystal structure of AGlc-M; Figure S2. Molecular packing in the crystal structure of AGlc-O; Figure S3. Molecular packing in the crystal structure of AGlc-M-LT; Figure S4. Calculated electrostatic potential and Hirshfeld surface for AGlc-M molecule; Figure S5. Molecular packing in the crystal structure of MGlc. *P*2_1_; Table S1: Selected bond lengths (Å) and torsion angles (°) in conformers of MGlc and AGlc.
