# Supplementary material for: Synthesis and Characterization of Biodegradable Polymers Based on Glucose Derivatives
Source: Materials (Basel). 2022 Dec 27;16(1):253. doi: 10.3390/ma16010253 (PMC9822088; doi:10.3390/ma16010253)
Supplement: Supplementary file 1 [file materials-16-00253-s001.zip › materials-2113327-supplementary.pdf]

materials

MDPI

Supplementary Materials

Synthesis and Characterization of Biodegradable Polymers Based on Glucose Derivatives

Single-Crystal X-ray Crystallography

Crystal data for **MGlc**: $C_{18}H_{24}O_{11}$, T = 293 K, M = 416.37 g/mol, monoclinic, space group $P2_1$, a = 9.729(1) Å, b = 6.264(1) Å, c = 17.351(2) Å, β = 93.24(1)°, V = 1055.7(2) Å^3, Z = 2, μ = 0.943 mm^{-1}, d_{calc} = 1.310 g/cm^3, 25234 reflections measured (4.55 ≤ θ ≤ 68.46°), 3871 unique (R_{int} = 0.0633) which were used in all calculations. The final R_1 was 0.035 (I>2σ(I)) and wR_2 was 0.1008 (all data).

Crystal data for **AGlc-M**: $C_{17}H_{22}O_{11}$, T = 293 K, M = 402.34 g/mol, monoclinic, space group $P2_1$, a = 5.821(1) Å, b = 13.970(3) Å, c = 12.769(3) Å, β = 100.06(2)°, V = 1022.4(4) Å^3, Z = 2, μ = 0.956 mm^{-1}, d_{calc} = 1.307 g/cm^3, 3844 reflections measured (3.52 ≤ θ ≤ 74.72°), 2897 unique (R_{int} = 0.015) which were used in all calculations. The final R_1 was 0.0977 (I>2σ(I)) and wR_2 was 0.3034 (all data).

Crystal data for **AGlc-O**: $C_{17}H_{22}O_{11}$, T = 293 K, M = 402.34 g/mol, orthorhombic, space group $P2_12_12_1$, a = 5.976(1) Å, b = 18.730(2) Å, c = 36.605(4) Å, V = 4097.2(9) Å^3, Z = 8, μ = 0.954 mm^{-1}, d_{calc} = 1.305 g/cm^3, 29892 reflections measured (4.33 ≤ θ ≤ 69.11°), 7277 unique (R_{int} = 0.0661) which were used in all calculations. The final R_1 was 0.0558 (I>2σ(I)) and wR_2 was 0.1767 (all data).

Crystal data for **AGlc-M-LT**: $C_{17}H_{22}O_{11}$, T = 120 K, M = 402.34 g/mol, monoclinic, space group $P2_1$, a = 14.813(4) Å, b = 13.566(6) Å, c = 15.550(5) Å, β = 110.08(4)°, V = 2935(2) Å^3, Z = 6, μ = 0.999 mm^{-1}, d_{calc} = 1.366 g/cm^3, 10342 reflections measured (3.56 ≤ θ ≤ 79.15°), 8261 unique (R_{int} = 0.0527) which were used in all calculations. The final R_1 was 0.2165 (I>2σ(I)) and wR_2 was 0.5654 (all data).

Table S1. Selected bond lengths (Å) and torsion angles (°) in conformers of MGlc and AGlc.

	MGlc	AGlc-**M**	AGlc-**O**	AGlc-**O**	AGlc-**M-LT**	AGlc-**M-LT**	AGlc-**M-LT**
Bond lengths (Å)			mol. A	mol. B	mol. A	mol. B	mol. C
C1–O1	1.413(3)	1.401(10)	1.401(6)	1.422(5)	1.38(3)	1.40(2)	1.45(2)
C1–O5	1.409(3)	1.394(11)	1.397(5)	1.397(6)	1.38(3)	1.37(2)	1.40(2)
C5–O5	1.432(2)	1.419(10)	1.434(6)	1.433(5)	1.41(2)	1.44(3)	1.41(2)
C2–O2	1.441(3)	1.448(10)	1.439(6)	1.436(5)	1.43(3)	1.43(2)	1.48(2)
C3–O3	1.441(3)	1.457(10)	1.442(5)	1.427(5)	1.50(3)	1.40(3)	1.47(3)
C4–O4	1.444(2)	1.436(9)	1.448(6)	1.433(5)	1.45(2)	1.46(3)	1.44(2)
C6–O6	1.444(3)	1.447(13)	1.426(7)	1.423(6)	1.45(3)	1.46(3)	1.49(3)
Torsion angles (°)							
Pyranose ring							

O5–C1–C2–C3	58.9(2)	51.6(9)	51.5(5)	55.7(5)	44(3)	54(2)	54(2)
C1–C2–C3–C4	-51.1(2)	-48.4(9)	-47.0(5)	-51.1(5)	-46(3)	-48(3)	-48(3)
C2–C3–C4–C5	48.9(2)	51.4(8)	51.2(5)	52.0(5)	51(3)	52(3)	50(3)
C3–C4–C5–O5	-53.6(2)	-58.8(9)	-60.9(5)	-55.7(5)	-57(2)	-57(3)	-58(2)
C4–C5–O5–C1	62.3(2)	65.7(10)	67.9(5)	61.3(5)	57(3)	65(3)	66(2)
C5–O5–C1–C2	-65.2(2)	-61.8(10)	-62.0(5)	-61.7(5)	-49(3)	-63(2)	-64(2)
substituents							
O5–C5–C6–O6	-48.6(2)	68.1(10)	66.3(6)	-59.2(5)	67(3)	66(2)	64(2)
C5–O5–C1–O1	179.9(2)	-177.8(7)	-176.3(4)	-178.2(4)	-172(2)	-172(2)	-179(2)
O5–C1–O1–C11	-99.4(2)	-78.0(11)	-84.2(6)	**-120.3(4)**	-71(3)	-83(3)	-71(2)
C1–O1–C11–C21	-172.6(2)	176.2(9)	-179.9(4)	-179.4(4)	174(2)	172(2)	175(2)
O1–C11–C21–C31	-5.1(6)	172.6(15)	174.1(7)	175.2(6)	174(3)	**5(4)**	167(2)
O5–C1–C2–O2	177.0(2)	171.6(6)	172.4(4)	174.3(3)	168(2)	172(2)	174(2)
C1–C2–O2–C12	114.8(3)	108.9(8)	111.0(5)	**135.5(4)**	108(2)	115(2)	108(2)
C2–O2–C12–C22	-168.5(3)	-177.1(8)	-176.9(4)	-178.3(4)	-175(2)	-177(2)	178(2)
C1–C2–C3–O3	-170.2(2)	-167.2(6)	-167.4(4)	-170.2(3)	-166(2)	-166(2)	-167(2)
C2–C3–O3–C13	-141.1(2)	-135.5(8)	**-170.2(3)**	**-101.6(5)**	-134(2)	-142(2)	-134(2)
C3–O3–C13–C23	-176.2(2)	173.4(4)	178.5(5)	178.4(4)	-180(2)	179(2)	170(2)
C2–C3–C4–O4	163.3(2)	167.4(7)	167.3(4)	169.6(4)	167(2)	169(2)	171(2)
C3–C4–O4–C14	93.3(2)	103.5(9)	99.7(5)	**115.3(4)**	103(2)	98(3)	104(2)
C4–O4–C14–C24	-179.4(2)	179.1(10)	177.8(5)	-177.8(4)	-178(2)	177(2)	177(2)

C3–C4–C5–C6	-174.5(2)	-176.3(7)	-178.3(4)	-176.5(4)	-175(2)	180(2)	-177(2)
C4–C5–C6–O6	73.5(2)	-173.6(6)	-175.2(4)	**63.0(5)**	-171(2)	-174(2)	-176(2)
C5–C6–O6–C16	-103.2(2)	108.1(10)	123.5(5)	**162.1(5)**	98(3)	124(2)	97(2)
C6–O6–C16–C26	-177.8(2)	-176.7(10)	**162.1(5)**	-174.4(5)	-179(3)	-179(2)	-174(2)

Molecular Structure

The anomeric C1–O1 bond length is shorter than all the exocyclic acetal C–O but longer on average by 0.015 Å, compared with the endocyclic C1–O5 bonds. However, both of them are shorter than the C5–O5 bond. This is in agreement with the C–O bond length distribution observed in some other β-anomers of pyranoses (Hayes et al., 2016; Baddeley et al., 2003; Root et al., 2002; Miler-Srenger et al., 1990). Due to the back-donation of the lone-pair electrons, the O5 atoms would have a partial positive charge, causing dipole attraction between the carboxy and pyranic oxygens. As confirmed by the O1–C1–O5–C5 and O5–C1–O1–C11 torsion angles (Table 1S), the conformation about the anomeric bonds is +*ap*, **–***ac* (**MGlc**), **–***ap*, **–***ac* (**AGlc-O –** mol B) or **–***ap*, **–***sc* (**AGlc-M**, **AGlc-O –** mol A, **AGlc-M-LT**). The acetyl O6 is ±*synclinal* (*gauche*) with respect to O5.

In all molecules, the pyranose ring has a slightly distorted ($^4C_1$) chair conformation. The distortion of the rings from the ideal chair geometry can result from the repulsion of 2,3- and 3,4-*O*-acetyl groups.

The molecular overlay of **AGlc-M**, **AGlc-O** and **AGlc-M-LT** (Fig. 7) indicates that the essential difference between the conformers in the polymorphs is in the relative orientation of the acryl and acetyl groups with respect to the central pyranose. The reorientation of the acryl group can be described by the O5–C1–O1–O11 torsion angle which adopts the values: **–**78(1)° (**AGlc-M**), **–**84.2(6)° (**AGlc-O –** mol A), **–**120.3(4)° (**AGlc-O –** mol B), **–**71(3)° (**AGlc-M-LT –** mol A), **–**83(3)° (**AGlc-M-LT –** mol B), **–**71(2)° (**AGlc-M-LT** – mol C). Another important difference is in the geometry of the acryl unit, which adopts the *cis* conformation (methylene C31 with respect to carboxyl O1) in **AGlc-M-LT –** mol B and *trans* conformation in all remaining structures (Table S1).

Crystal Structure

Due to the chemical composition of molecules, there are various intermolecular C–H···O, C–H···π and π···π contacts in the studied crystals (Fig. S4). The presence of numerous acceptors and highly polarized C–H groups stimulates the formation of weak hydrogen bonds. Among them, the C–H···$O_{carbonyl}$ and C–H···O_{gluc} contacts seem to play the dominant role in the stabilization of the resulting structures (Figs S1-S5). Though the vast majority of acetal O-atoms are excluded from hydrogen bonding.

The main supramolecular motifs in all detected forms are folded molecular stacks, composed of translation-related molecules. In crystals **MGlc** and **AGlc-M,** they are propagated along the b and a axis, respectively. Stabilization of the motifs is provided by an extensive network of C–H···O bonds, involving both the glucopyranose as well as acetyl and meth/acryl groups.

The 1D motifs (2_1-axis related) are linked to the 3D architecture by C–H···O contacts, however, without glucopyranose ring atoms.

In the case of the crystal **AGlc-O, the** separation of symmetry-independent molecules is clearly visible. The molecules A and B (assembled into the pseudo-centrosymmetric

dimers) are involved in the formation of alternating stacks running along the a axis. The adjacent 2_1-axis related motifs are arranged into distinct (001) layers. Despite the great similarity in the relative orientation of the molecules, the hydrogen-bonding patterns in each 1D motif are quite different. In low-temperature polymorph **AGlc-M-LT,** the (101) stacks are built of alternating A-, B-, and C-type molecules.

The lack of specific, strong hydrogen bonds directing the supramolecular organization towards a given structure, together with conformational flexibility resulting from rotation around C–O bonds, favors polymorphic modifications.

AGlc-M *P*2_1 Z′ = 1 view down the *a* axis

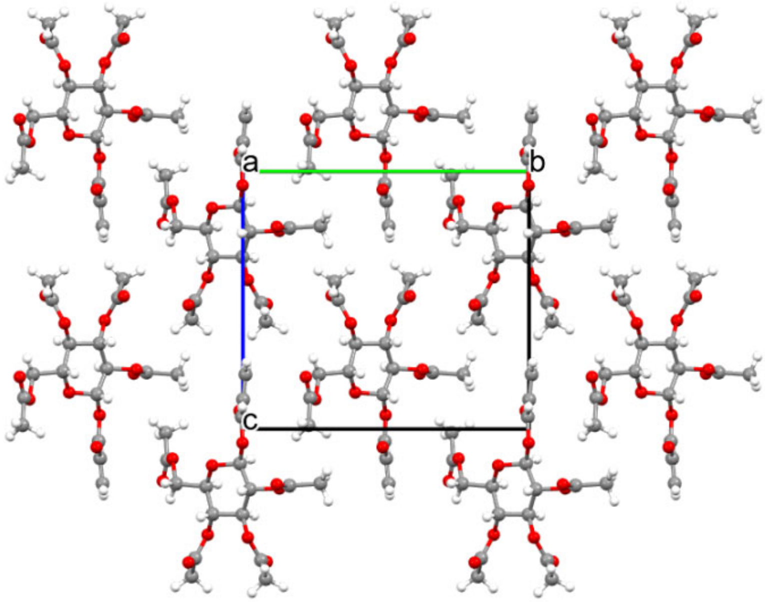

view down the *c* axis

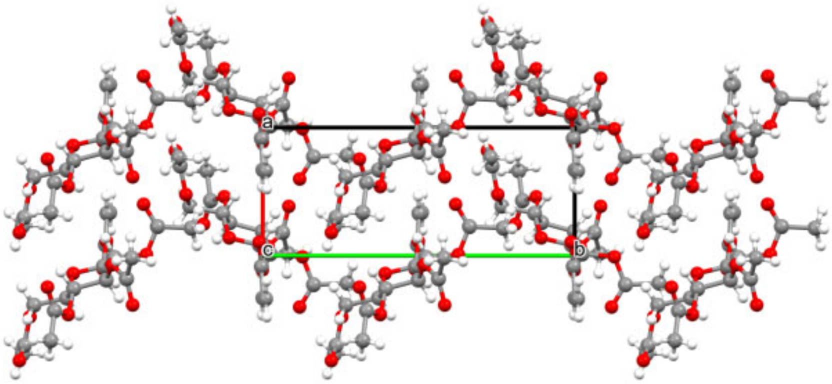

Figure S1. Molecular packing in crystal structure of **AGlc-M**.

AGlc-O $P2_12_12_1$ $Z' = 2$ view down the *a* axis

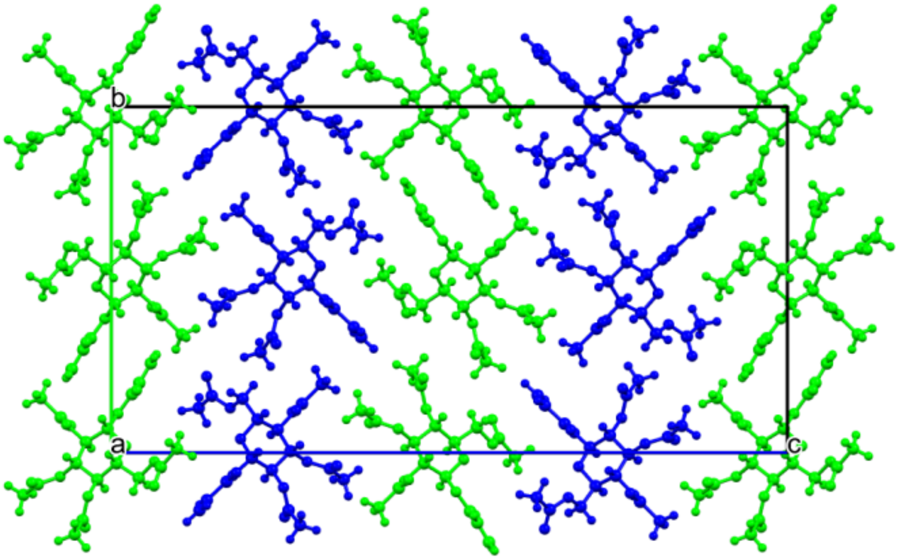

view down the *b* axis

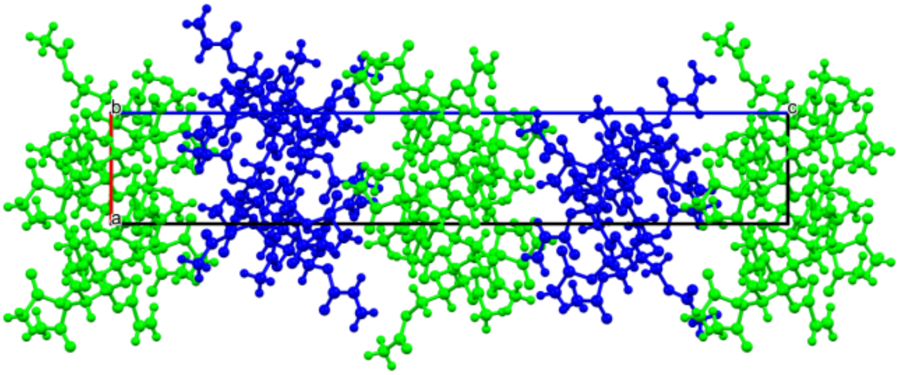

Figure S2. Molecular packing in crystal structure of **AGlc-O**.

AGlc-M-LT	$P2_1$	$Z' = 3$	view down the *a* axis

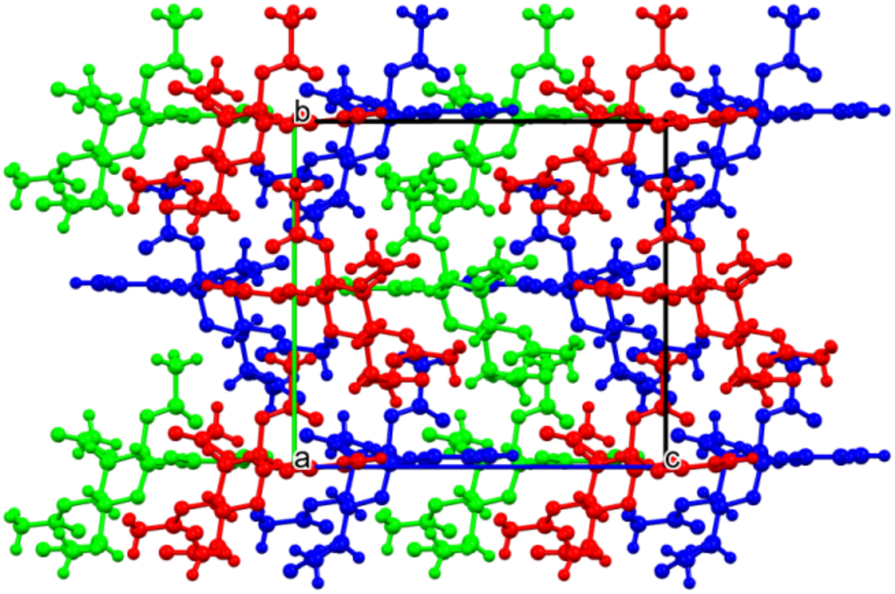

view down the *b* axis

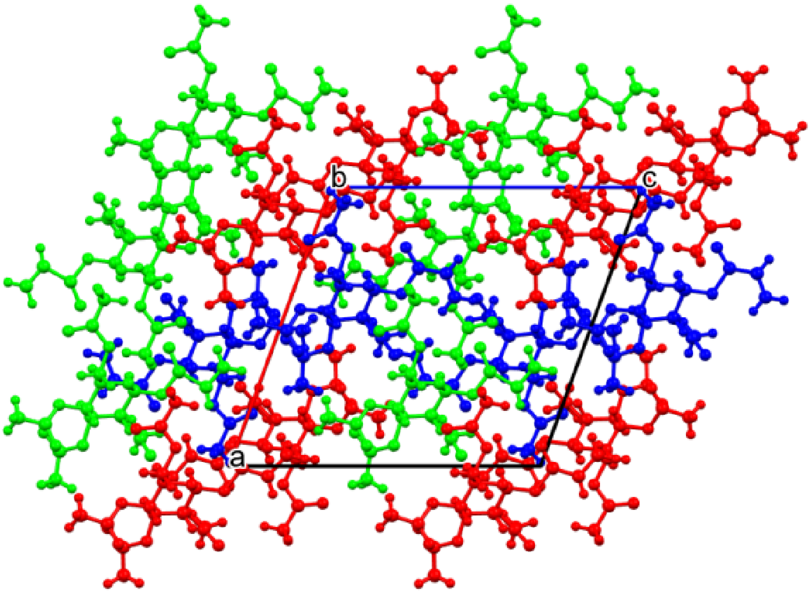

Figure S3. Molecular packing in crystal structure of **AGlc-M-LT**.

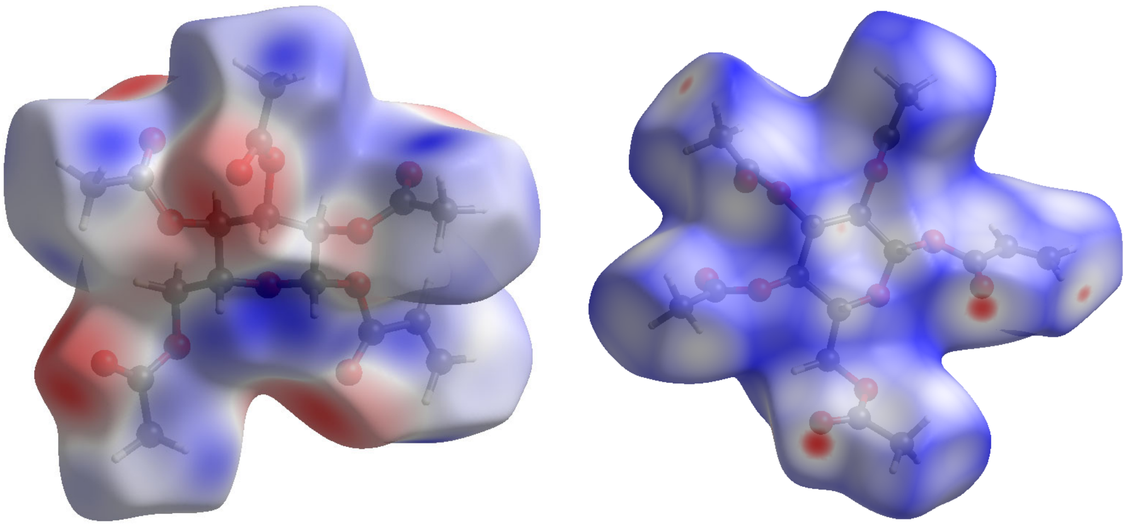

AGlc-M: electrostatic potential	AGlc-M: Hirshfeld surface d_{norm} plot
(min -0.0662 au, max 0.0395 au)	(min -0.170 Å, max 1.362 Å)

Figure S4. Calculated electrostatic potential and Hirshfeld surface for AGlc-**M** molecule.

view down the *b* axis

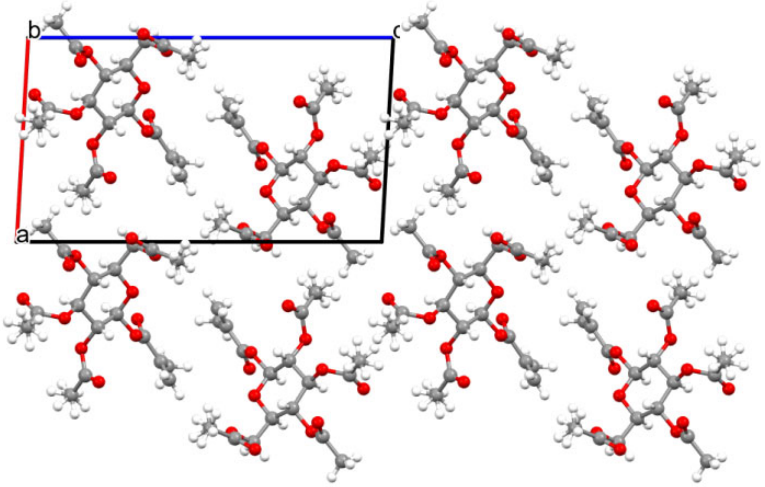

view down the *a* axis

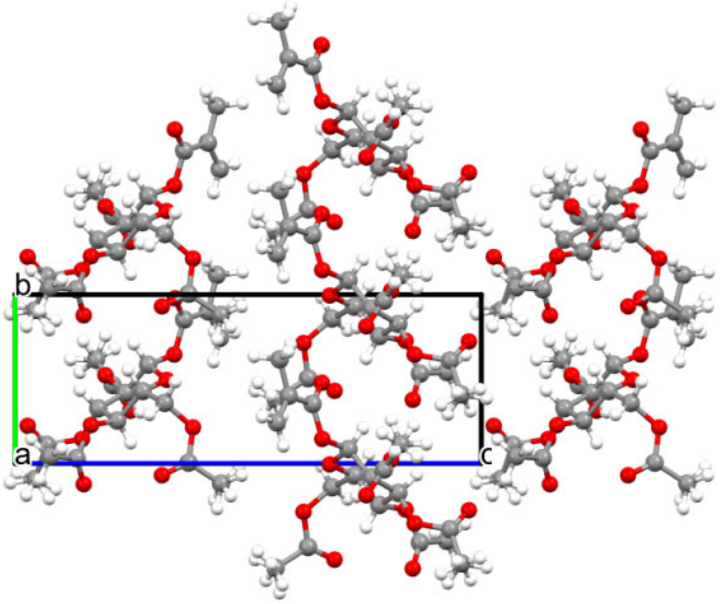

Figure S5. Molecular packing in crystal structure of **MGlc**. $P2_1$
